# Vegetation expansion in the subnival Hindu Kush Himalaya

**DOI:** 10.1111/gcb.14919

**Published:** 2020-01-09

**Authors:** Karen Anderson, Dominic Fawcett, Anthony Cugulliere, Sophie Benford, Darren Jones, Ruolin Leng

**Affiliations:** ^1^ Environment and Sustainability Institute University of Exeter Cornwall UK; ^2^ Department of Geography University of Exeter Cornwall UK; ^3^ Department of Environment and Resources Lanzhou University Lanzhou Gansu China

**Keywords:** ecohydrology, Himalaya, Landsat, NDVI, remote sensing, subnival, time series

## Abstract

The mountain systems of the Hindu Kush Himalaya (HKH) are changing rapidly due to climatic change, but an overlooked component is the subnival ecosystem (between the treeline and snow line), characterized by short‐stature plants and seasonal snow. Basic information about subnival vegetation distribution and rates of ecosystem change are not known, yet such information is needed to understand relationships between subnival ecology and water/carbon cycles. We show that HKH subnival ecosystems cover five to 15 times the area of permanent glaciers and snow, highlighting their eco‐hydrological importance. Using satellite data from the Landsat 5, 7 and 8 missions, we measured change in the spatial extent of subnival vegetation from 1993 to 2018. The Landsat surface reflectance‐derived Normalized Difference Vegetation Index product was thresholded at 0.1 to indicate the presence/absence of vegetation. Using this product, the strength and direction of time‐series trends in the green pixel fraction were measured within three regions of interest. We controlled for cloud cover, snow cover and evaluated the impact of sensor radiometric differences between Landsat 7 and Landsat 8. Using Google Earth Engine to expedite data processing tasks, we show that there has been a weakly positive increase in the extent of subnival vegetation since 1993. Strongest and most significant trends were found in the height region of 5,000–5,500 m a.s.l. across the HKH extent: *R* ^2^ = .302, Kendall's *τ* = 0.424, *p* < .05, but this varied regionally, with height, and according to the sensors included in the time series. Positive trends at lower elevations occurred on steeper slopes whilst at higher elevations, flatter areas exhibited stronger trends. We validated our findings using online photographs. Subnival ecological changes have likely impacted HKH carbon and water cycles with impacts on millions of people living downstream, but the strength and direction of impacts of vegetation expansion remain unknown.

## Graphical abstract

Using freely available data from the NASA Landsat missions 5, 7 and 8, we showed that subnival vegetation across the Hindu Kush Himalayan region has expanded since 1993. This expansion is likely to have impacts on carbon cycling and hydrology with impacts for millions of people living downstream. Further work is needed to quantify the impacts of this high altitude vegetation change.

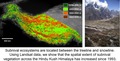

## 1 INTRODUCTION

Mountain systems are amongst the most dynamic on Earth and are particularly sensitive to climatic change (Dolezal et al., 2016). The Hindu Kush Himalayan (HKH) region (the ‘third pole’) covers 4.2 million km^2^ and feeds the 10 largest river systems in Asia (Bajracharya et al., 2015), supplying ~1.4 billion people with water (Bolch et al., 2012; Immerzeel, Beek, & Bierkens, 2010). HKH warming rates are higher than the global average (Pachauri et al., 2014; Peng, Piao, Ciais, Fang, & Wang, 2010) and recent work concludes with high confidence that snow‐covered areas and snow volumes will decrease across most HKH regions over coming decades, in response to climatic change (Bolch et al., 2019). For these reasons, most scientific work in the HKH region has focused on understanding the state and fate of glaciers (Bolch et al., 2012; Brun, Berthier, Wagnon, Kääb, & Treichler, 2017; Shannon et al., 2019), changes in glacial resources (Immerzeel et al., 2010; Kääb, Berthier, Nuth, Gardelle, & Arnaud, 2012; Kehrwald et al., 2008), hydrological risks (Shrestha et al., 2010; Worni, Huggel, & Stoffel, 2013) or monsoon‐driven run‐off dynamics (Armstrong et al., 2018; Thayyen, Gergan, & Dobhal, 2005). Ecological changes at high altitude have been comparatively overlooked, despite widespread understanding of the coupling of ecology and hydrology across spatial and temporal scales (Fatichi, Pappas, & Ivanov, 2016). The urgency with which this knowledge gap needs to be filled is evidenced by climate models showing how the spatial extent of temperature‐limited ecosystems in the HKH will reduce over the next 50–100 years, making more space available for vegetation expansion in the future (Keenan & Riley, 2018).

The subnival zone, which lies above the treeline and below the permanent snowline, is the most poorly studied vegetation zone in the HKH due to its inaccessibility and high altitude. In this region, ecological responses to climate change are likely to be multidimensional, species‐specific and spatially variable (Dolezal et al., 2016) with strong potential for feedbacks between plant cover, carbon, snow and hydrological processes to occur, as evidenced by relatively advanced work from Arctic systems (Myers‐Smith et al., 2011, 2015; Myers‐Smith & Hik, 2013). In the HKH, feedbacks have been shown to exist between climate and plant phenology (Chen, Zhu, Wu, Wang, & Peng, 2011) and between shrub cover and snowpack dynamics (Hu et al., 2009). There is, meanwhile, evidence of shrub encroachment into Himalayan grasslands at high altitude (Brandt, Haynes, Kuemmerle, Waller, & Radeloff, 2013; Chophyel, 2009; Qiu, 2016; Wangchuk, Gyaltshen, Yonten, Nirola, & Tshering, 2013), and warming‐driven geographical range shifts in endemic plant species (Dolezal et al., 2016; Telwala, Brook, Manish, & Pandit, 2013). Spatial ecological changes across the full extent of the HKH subnival zone now need scientific attention as we approach, and pass, peak non‐renewable water (Gleick & Palaniappan, 2010; Huss & Hock, 2018; Jones, Harrison, Anderson, & Whalley, 2019), and yet across the HKH extent, there is no basic understanding of current subnival vegetation distribution, and no information on rates of change over past decades.

A starting point from which to evaluate the present status, geographical distribution and historical trend in vegetation change across the HKH is to exploit freely available medium resolution (30 m pixel size) satellite data. Whilst this seems relatively straightforward in principle, there are practical challenges caused by the huge data volumes needed to evaluate changes in vegetation cover over thousands of square kilometres and over multi‐decadal timescales. Classical remote sensing workflows where satellite data are downloaded from online repositories and then processed locally on computers, are computationally‐ and time‐expensive which prohibits analyses on an area this large. To address this challenge, we exploited the unique freely accessible cloud‐processing capabilities of the new Google Earth Engine (GEE) platform, which houses public data catalogues for the whole of the Landsat archive (Gorelick et al., 2017). We focus on answering two questions:

1. What is the *extent* of the subnival zone?
2. Has the *spatial extent* of subnival vegetation changed and, if so, at what *rate* and *where*?

With regard to answering question 2, our work was concerned with evaluating evidence for subnival system transition from bare ground or sparsely vegetated ground to vegetated ground; not change in species composition.

## 2 METHODOLOGY

### 2.1 Study system: The subnival zone

**Figure 1:**
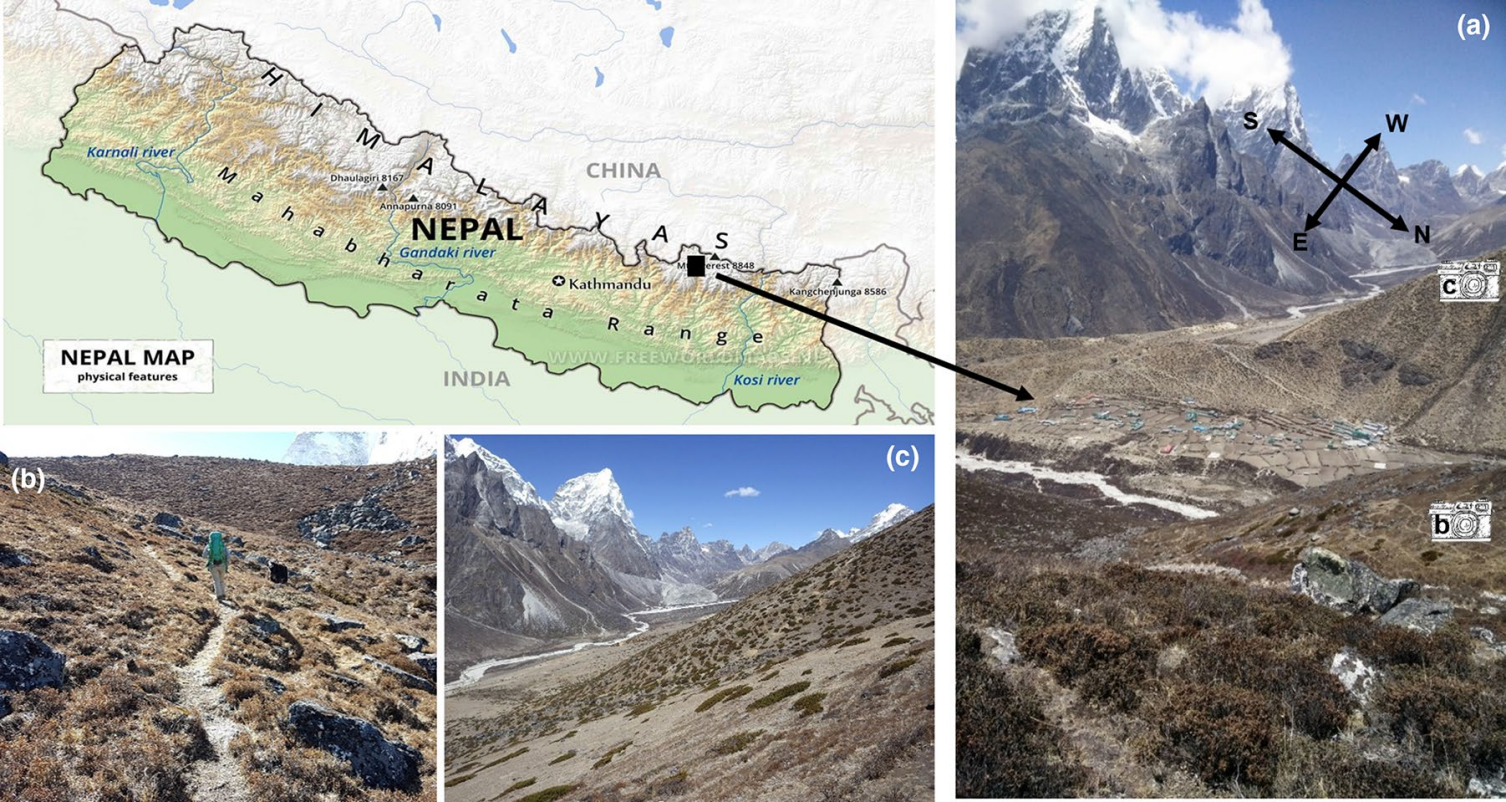
Photographs (by authors K. Anderson; D Jones) of typical subnival ecosystems in the Sagarmatha National Park, Nepal. (a) A view across the valley with the village of Dingboche (4,410 m a.s.l.) below, taken from an elevation of around 4,900 m a.s.l. Viewpoints (b) and (c) are overlaid. (b) The view east, looking towards the base of Ama Dablam, at around 4,700 m a.s.l., whilst (c) is the view towards Chola Glacier (beneath Cholatse peak) at 4,700 m a.s.l. looking west. Clearly visible are dwarf shrubs including the species *Rhododendron anthopogon* [Colour figure can be viewed at http://www.wileyonlinelibrary.com]

Ecosystems in the HKH region are diverse due to the variety of complex climatic conditions, alongside stark variations in altitude (Dorji, Olesen, Bøcher, & Seidenkrantz, 2016) and slope aspect (Anthwal, Bhatt, Nautiyal, & Anthwal, 2011). The treeline in the Himalaya is typically found at approximately 4,000 m above sea level (m a.s.l.; Gaire, Koirala, Bhuju, & Borgaonkar, 2014) although certain tree species have been shown to grow at altitudes of up to 4,900 m a.s.l. (e.g. in Tibet; Miehe, Miehe, Vogel, Co, & La, 2007). The treeline of the Himalayan Birch (*Betula utilis*), a particularly abundant species, is found between 3,900 and 4,150 m a.s.l. (Liang, Dawadi, Pederson, & Eckstein, 2014). Above the treeline exists a high‐altitude region colonized by herbaceous plants and dwarf shrubs, the annual growth phase of which is initiated synchronously with seasonal temperature rise and snow melt (Pangtey, Rawal, Bankoti, & Samant, 1990). A useful description of the major Himalayan plant zones is provided by Ives and Messerli (1989), who describe the upper timberline as occurring between 4,000 and 4,500 m a.s.l., above which a rhododendron‐shrub belt grades into alpine meadows, with a ‘subnival belt of extensive bare ground and scattered dwarf plants, mosses and lichens’. They describe the 5,000–5,500 m a.s.l. elevation zone as being characterized by permanent ice and snow with steep rocky outcrops. In this work, we use the upper elevational limit of *B. utilis* (i.e. 4,150 m a.s.l.) to define the start of the subnival zone, where shrub species such as *Rhododendron anthopogon* (a dwarf shrub known locally in Nepal as *sunpaati*, or colloquially as incense rhododendron; E. Byers, personal communication, May 2, 2017) become more abundant. Typical Himalayan subnival ecosystems are shown in Figure 1, using photographs captured at above 4,700 m a.s.l. in Nepal. These evidence the broad coverage of *R. anthopogon* shrubs and other dwarf grasses and shrubs at high elevation.

### 2.2 Satellite data analysis

We utilized data from the NASA Landsat satellite mission, which provides long time‐series (1972–present) medium resolution (~30 m pixels) imaging data over the region (Cohen & Goward, 2004; Roy et al., 2014). Such data have a long history of use for monitoring land cover change (Aplin, 2004) including in mountainous regions (Cingolani, Renison, Zak, & Cabido, 2004; Helmer, Brown, & Cohen, 2000; Levin, Shmida, Levanoni, Tamari, & Kark, 2007; White, Running, Nemani, Keane, & Ryan, 1997), in areas of short‐sward vegetation (Chopping et al., 2008; Goslee, Havstad, Peters, Rango, & Schlesinger, 2003; Laliberte et al., 2004; Skowno et al., 2017; Tian, Brandt, Liu, Rasmussen, & Fensholt, 2017), and at sites within the HKH (Brandt et al., 2013). Landsat missions also offer long time‐series data with a similar sensor, a significant benefit because some work has shown the lack of consistency when comparing vegetation metrics between different sensors (Abuzar, Sheffield, Whitfield, O'connell, & Mcallister, 2014). Indeed, a recent study suggested that freely available data from satellites such as NASA's Landsat were ‘remarkably congruent’ for broadscale monitoring of shrub encroachment in African savannahs (Marston, Aplin, Wilkinson, Field, & O'regan, 2017), which have short‐stature, patchy vegetation structurally similar to that within the HKH subnival zone. At 30 m pixel resolution, Landsat data offer considerable benefits for vegetation time‐series analysis in mountainous areas, particularly as compared to other satellite‐derived vegetation products. For example, the GIMMS 3g fAPAR product used by Keenan and Riley (2018) for evaluation of global cold climate ecosystem change has a spatial resolution of 8 km, such that, across the HKH, one would find significant variations in elevation and ecology over the space of a single pixel. Corroborating this, recent work by Fassnacht, Schiller, Kattenborn, Zhao, and Qu (2019) evidences the lack of suitability of coarse‐grained 500 m resolution MODIS vegetation products for mapping changes in high altitude vegetation on the Tibetan Plateau. In contrast, they showed that Landsat data could allow for ‘detailed spatial patterns of land cover changes’ to be identified (Fassnacht et al., 2019). Importantly, Fassnacht et al. (2019) corrected for radiometric differences between Landsat 7 Enhanced Thematic Mapper (TM) and Landsat 8 Operational Land Imager (OLI) detectors using published coefficients from Roy et al. (2016).

Our study analysed data from three Landsat missions (5, 7 and 8) to evaluate time‐series dynamics in the presence/absence of subnival vegetation cover over the 26 year period from 1993 to 2018. Data from the period prior to 1993 were not included due to patchy time‐series coverage and because of the complexity of issues associated with integrating data from earlier missions (i.e. Landsat 1–4). We subsequently split the analysis to study vegetation changes across three regions encompassing different spatial extents: (a) Sagarmatha (Everest) National Park, Nepal; (b) the national extent of Nepal; and (c) the entire HKH region. For each of these regions, we measured vegetation trends in four elevation bands:

- H1 (4,150–4,500 m a.s.l.);
- H2 (4,500–5,000 m a.s.l.);
- H3 (5,000–5,500 m a.s.l.);
- H4 (5,500–6,000 m a.s.l.).

Subsequent sections explain how the GEE methodology was implemented for each of these in turn.

#### 2.2.1 Sagarmatha National Park area, Nepal (‘P140‐R40/41 region’)

**Figure 2:**
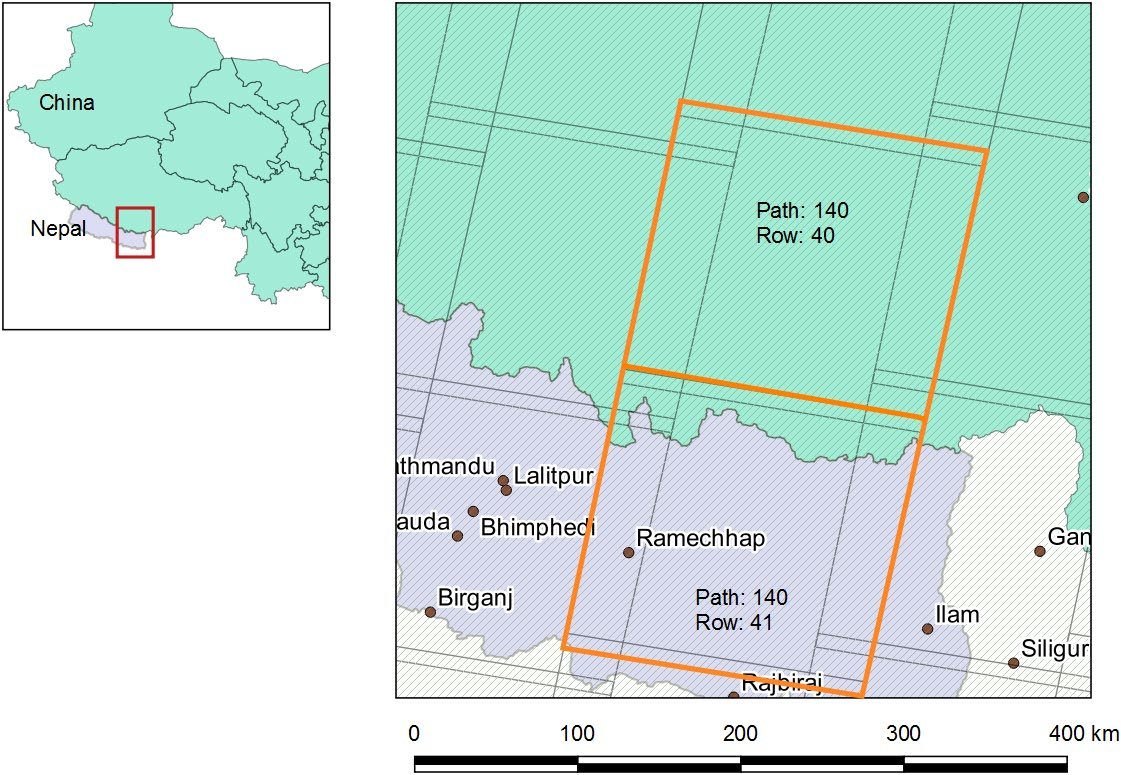
Location of the P140‐R40/41 region of interest, which utilized data from Landsat path 140, rows 41 and 42 on the Nepal/Tibet border [Colour figure can be viewed at http://www.wileyonlinelibrary.com]

The first part of the analysis focused on analysing time‐series change in two tiles of Landsat data (path 140, rows 40 and 41; Figure 2), covering a region on the Nepal/Tibet border centred on Mount Everest. This was chosen for initial exploration for several reasons. This region contains the greatest elevation range in the Himalayas, and includes areas of glacierized (i.e. ice‐covered) terrain and recently deglaciated terrain (including the Khumbu region, south of Everest; Byers, 2007). In the northernmost part of the image extent are areas on the Tibetan plateau exceeding 6,000 m a.s.l. where previous reports have suggested vegetation change is occurring rapidly (e.g. Qiu, 2016). Furthermore, these data also cover a broad range of climatic conditions including precipitation gradients caused by winds and orographic barriers (Bookhagen & Burbank, 2006). The area thus provides a good starting point for investigating vegetation change because it is a microcosm of the range of conditions (particularly elevation and aridity) experienced across the wider HKH. Figure 2 shows the location of the two tiles of Landsat data analysed in this initial phase, and throughout the rest of this paper, we refer to this region as P140‐R40/41.

#### 2.2.2 National extent of Nepal

The second part of the analysis focused on time‐series change across the areal extent of the country of Nepal. For this, a KMZ shapefile delineating the boundary of Nepal was uploaded into GEE and used to constrain the analysis.

#### 2.2.3 HKH‐wide analysis

**Figure 3:**
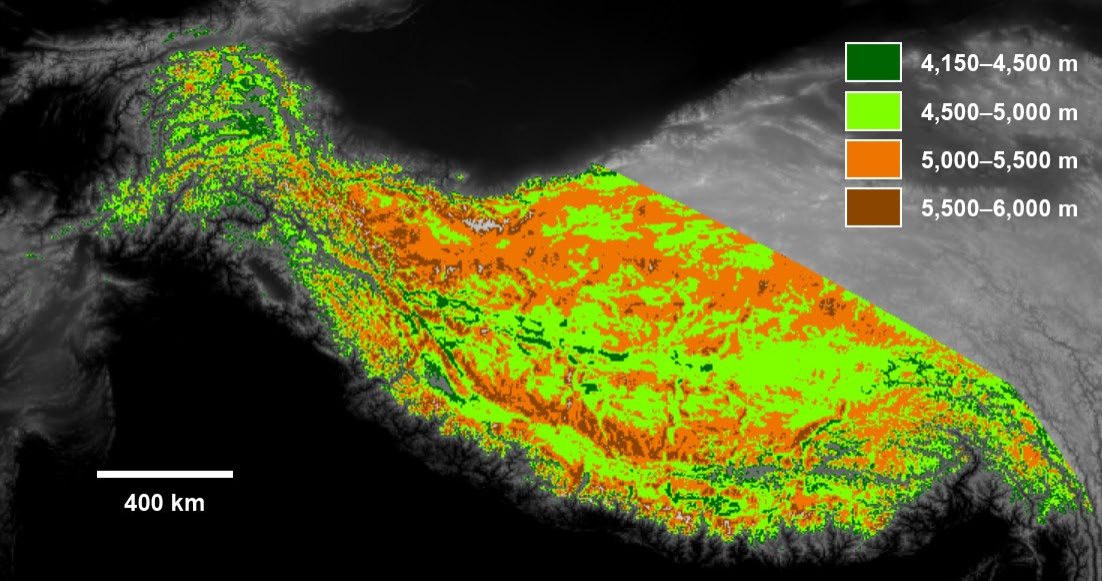
Defining the Hindu Kush Himalaya‐wide sample region, highlighting elevation bands explored in the rest of the paper [Colour figure can be viewed at http://www.wileyonlinelibrary.com]

Google Earth Engine allocates users a fixed processing capacity, so to be able to measure change over the entire HKH, a random sampling method using regions of interest (ROIs) was necessary. We defined 100 circular ROIs with a 5 km radius and randomly deployed these within each of the four height bands previously described. The total area covered by the ROIs that were used to sample the satellite data record equalled 31,416 km^2^ (overlap of ROIs and height bands not taken into account). Figure 3 shows the spatial distribution of the different height bands across the HKH sampled using the circular ROIs.

### 2.3 GEE workflows

#### 2.3.1 Permanent snow to seasonal snow cover calculations

To answer question 1 (*What is the extent of the HKH subnival zone?*), the freely available moderate resolution imaging spectrometer (MODIS) fractional snow product (Hall, Riggs, Salomonson, Digirolamo, & Bayr, 2002) was used and analyses were run at two scales: first for the P140/R40‐41 region, and second, for the country of Nepal. We selected recent years (2013–2017) to generate a product describing the median snow‐covered area in late summer (August and September), when the snow cover is at a minimum (determined using: http://geoapps.icimod.org/HKHSnowCover/) averaged over a 1 km pixel scale. This output was used to represent permanent snow‐covered areas for the ROI. The permanent snow‐covered area was combined with the Randolph Glacier Inventory (RGI; Pfeffer et al., 2014; which was at a finer spatial resolution) to estimate the spatial extent of permanent snow and ice cover. This was compared to the extent of the entire subnival zone, represented by the total area above 4,150 m a.s.l. using the Shuttle Radar Topography Mission (SRTM) 30 m gridded data set as a measurement of height above mean sea level (Farr et al., 2007). To estimate the total area of permanent snow and ice, the snow area on glaciers was subtracted from permanent snow cover + permanent ice cover. The final ratio between the subnival habitat area which can contain seasonal snow and ice, and permanent snow and ice were calculated by first, subtracting the total area of permanent snow and ice from the total land area above 4,150 m a.s.l., and then dividing the two values.

#### 2.3.2 Landsat surface reflectance product procedure for vegetation fractional cover determination

To answer question 2 (Has the *spatial extent* of subnival vegetation changed; and if so, at what *rate* and *where*?), Landsat Tier 1 surface reflectance (SR) data processed by USGS were used. The SR products contain pixel quality attribute information resulting from the atmospheric correction process. Likely cloud, snow and shadow pixels are flagged and can be used to mask undesirable data, whilst the remaining SR values are expected to be more consistent than standard top of atmosphere radiance products, because differences in the atmospheric composition are taken into account. However, USGS caution that SR products could be adversely affected by hyper‐arid or snow‐covered conditions, which exist in certain regions of the area studied here (USGS, 2018). We needed a simple vegetation product to allow vegetated pixels to be discriminated from unvegetated pixels, but a complex land cover analysis or retrieval of biophysical vegetation properties was not required. For this reason, a simple and widely used index, the Normalized Difference Vegetation Index (NDVI), was chosen. Other studies have shown that NDVI is a relatively robust indicator of green biomass even in sparsely vegetated systems (Gamon et al., 1995), and that specifically, Landsat TM and enhanced TM (ETM+) derived NDVI delivered consistent data to surface‐measured NDVI in Arctic systems (Pattison, Jorgenson, Raynolds, & Welker, 2015), which have similar low‐stature plants. Recent work by Fassnacht et al. (2019) on the Tibetan plateau has also shown the suitability of Landsat NDVI products for measuring high altitude vegetation change. For these reasons, we argue that NDVI derived from SR data will produce a robust presence/absence indicator for vegetation in subnival HKH systems. The description of the applied workflow is given in the below paragraph with key factors marked in italic.

First, data from *Landsat 5, 7 and 8 Tier 1 SR image collections* were filtered to extract data from specific post‐monsoon months *(October and November).* This period in the post‐monsoon season is when the HKH does not receive a significant volume of rainfall and when cloud and snow cover are expected to be at an annual minimum (Bookhagen & Burbank, 2006). By constraining the analysis to this short post‐monsoon period, phenological differences between individual acquisitions were also limited. Pixels flagged as cloud, snow or shadow in the per‐pixel quality assessment (QA) layer were then masked in the individual images. To verify whether there were any significant changes in snow fractional cover over time over the years analysed, a snow cover product based on pixels flagged as snow within the Landsat QA flags was also generated to derive a fraction of snow‐covered pixels per year for the three spatial extents considered throughout this paper.

Landsat 5, 7 and 8 collections were then merged and NDVI was computed for each image in the collection. To composite multiple images available for the observed time period and fill regions of missing data due to cloud cover, an *annual vegetation index average* was generated using the median NDVI value per pixel from image sets captured within a single season. Finally, an *aspect mask* was built by computing aspect based on the *SRTM global digital elevation model (DEM) at 30 m resolution*, but the analysis was rescaled to 90 m resolution during the pixel counting phase (to calculate green fraction, described below). We masked all values <*45*°* and >315*° to remove north facing slopes that were likely to be shaded by neighbouring topography (i.e. causing the most significant bidirectional reflectance distribution function [BRDF] effects) and these were excluded from all subsequent analyses. The aspect and elevations outside the height band analysed (using SRTM DEM data) were masked before summing the total number of unmasked pixels as well as the number of unmasked pixels with NDVI values above a 0.1 threshold and deriving the ‘*green fraction*’ per year by dividing the number of pixels *with an NDVI > 0.1* by the total number. An NDVI > 0.1 threshold was chosen to indicate the presence of vegetation—set at a low level due to the potential spatial sparsity of high altitude vegetation (Figure 1). The same procedure was used for generating *green fractions per year* for both regional analyses (P140‐R40/41, Nepal national extent) and to ROIs over the entire HKH.

### 2.4 Considerations surrounding Landsat 8 OLI to Landsat 7 ETM+ correction

Landsat 5 and Landsat 7 both use a ‘thematic mapper’ type sensor with very similar band positions and widths. Claverie, Vermote, Franch and Masek (2015) showed that Landsat SR products as used here were comparable between mission 5 and 7, with the exception of the blue band (not used in NDVI derivation). In contrast, the OLI sensor on board Landsat 8 differs from these previous missions: it still offers a 30 m spatial resolution, but the bands are spectrally narrower and cover different spectral ranges (Roy et al., 2016). To be more specific, blue, green and red Landsat 8 OLI band spectral response functions intersect with 82.76%, 71.08% and 60.63% of the corresponding Landsat 7 ETM+ band spectral response functions (Roy et al., 2016). Critically, for derivation of NDVI, which uses a near infra‐red (NIR) and red band, the L8 OLI's NIR band (centred on 850 nm) misses a water absorption feature that occurs in the corresponding ETM+ NIR band (Irons, Dwyer, & Barsi, 2012; Roy et al., 2016). These differences can impact the quality of comparisons between L7 and L8: Holden and Woodcock (2016) report that Landsat 8 is ‘consistently darker in red, green and blue bands’ compared to Landsat 7, and Roy et al. (2016) report that OLI NDVI was positively biased relative to ETM+ NDVI. Biases reported by Roy et al. (2016) were lowest with NDVI derived from the Landsat SR product, as used in our study. They report an average difference of +0.0165 (4.86%) between OLI and ETM+ derived NDVI, with minimal biases at NDVI values around the 0.1 threshold used here.

In GEE, a further consideration is that the Landsat 7 and 8 SR products available use slightly different algorithms for atmospheric correction and calibration to SR: Landsat 8 uses a scheme called ‘land surface reflectance code (LaSRC)’ (described in Li, Roy, Zhang, Vermote, & Huang, 2019; Vermote, Justice, Claverie, & Franch, 2016) whilst Landsat 7 data are calibrated using the ‘landsat ecosystem disturbance adaptive processing system (LEDAPS)’ scheme (Masek et al., 2006). Both LaSRC and LEDAPS use the same 6SV radiative transfer code (Vermote, Tanre, Deuze, Herman, & Morcette, 1997) with the primary difference being in the way that the aerosol content in the atmosphere is retrieved.

For robustness, we applied the L7/L8 correction method described by Roy et al. (2016) to our data prior to statistical analysis. This calibration is robust since it was developed using 59 million corresponding sensor observations extracted from 6,317 Landsat‐7 ETM+ and Landsat‐8 OLI images over the United States in winter and summer seasons (Roy et al., 2016). Alpine and high altitude vegetation with similar traits to the plants found in the HKH will be represented in this calibration model owing to the presence of high mountain systems across the conterminous United States. Fassnacht et al. (2019) have also applied the Roy et al. (2016) calibration to long time‐series analysis of vegetation on the Tibetan plateau further highlighting its suitability for our work in the HKH. From Roy et al. (2016), we used the parameters from their ordinary least squares (OLS) model, for converting OLI to ETM+ SR data, (*R* ^2^ = .926; *p* < .0001; Equation 1):

(1) ETM+=0.0029+0.9589OLI.

Remaining uncertainties which will not be accounted for by this approach would be BRDF and topographic effects not removed by the aspect masking (see Section 2.3.2), and aerosol differences not removed by the SR quality masking. Also, if pixels contained non‐vegetated dry terrain, the aerosol retrieval for LEDAPS and LaSRC could be quite different, which would manifest in blue, green and red band LEDAPS (Landsat 7) versus LaSRC (Landsat 8) differences (D. Roy, personal communication, 2019). To test the extent to which the latter effect impacted on our data, we used a glacial lake outburst flood scar near Panboche Nepal, which has remained unvegetated since the flood occurred in 1977 (Buchroithner, Jentsch, & Wanivenhaus, 1982) and is thus suitable for testing bare ground‐related biases. We measured the trend in Roy et al. (2016)‐corrected, SR‐based NDVI through the 1993–2018 time series. The results showed a non‐significant time‐series trend (see Figures S1 and S2; Table S1) so from hereon, we assumed bare ground‐related biases to be of minimal concern.

### 2.5 Statistical analysis

Time‐series trend analysis was undertaken using OLS regression (time vs. green fraction). Additionally, for robustness, a Mann–Kendall trend test, executed in R (package: Kendall v2.2, R version: 3.4.3), was applied. This additional trend analysis was undertaken since we noticed inter‐annual variability in some of the satellite time‐series data which could have impacted the results of the OLS regression. Mann–Kendall is accepted as a robust non‐parametric method to test for the presence and strength of monotonic trends in time series, and it exhibits a low sensitivity to short‐term variations. Results from both OLS and Mann–Kendall analyses are presented in this manuscript since they carry different information. The Kendall's *τ* parameter conveys information about the time‐series pattern, independently of the magnitude of change, whilst OLS coefficients allow trends to be modelled.

### 2.6 Validation

The lack of ecological studies across the subnival HKH means that there were no existing spatially distributed ecological data sets available for validation. However, there is a relatively new, rich resource of untapped ecological information in the form of publicly accessible photographs, available via Google. These *Photosphere* and *Streetview* images provide a means by which basic ecological data can be gathered (i.e. presence/absence of vegetation; and broad categorical classifications of the type of vegetation; e.g. grass/shrub). With the rise in mountain tourism, Streetview data are now provided for the full trekking path from Lukla to Everest Base Camp in Nepal (a 62 km long trail), for example. In order to validate one of the vegetation extent products (2017) for the P140‐R40/41 region, these shared, geolocated images available through Google Maps were used as a source of ecological data, specifically as a source of information about the presence/absence of vegetation and its broad typology. Digitizing ecological data from these images is a time‐consuming process because it requires manual visual enquiry of photographs, so we used a small crowdsourcing exercise to gather a set of observations for validation. A group of undergraduate students were trained in the methodology and they searched available images within predefined boundaries of the P140‐R40/41 region above an elevation of 4,150 m a.s.l. The location, presence, density and type of vegetation (classified into three main types: grass, shrub, moss) were recorded, as well as the date of the image capture. If the image contained no plants, participants could record an entry of ‘bare ground’. An online ‘Google sheets’ spreadsheet was used to collect the crowdsourced data and observations were checked for quality and filtered spatially to remove replicates by the authors. We supplemented the crowdsourced observations with a selection of our own field photographs captured during a fieldtrip to the Khumbu area of Nepal in April/May 2017. Observations of vegetation from in situ photographic data were then compared to satellite‐derived pixel attributes to determine the accuracy of the Landsat‐derived vegetation products.

## 3 RESULTS

### 3.1 What is the extent of the subnival zone?

**Figure 4:**
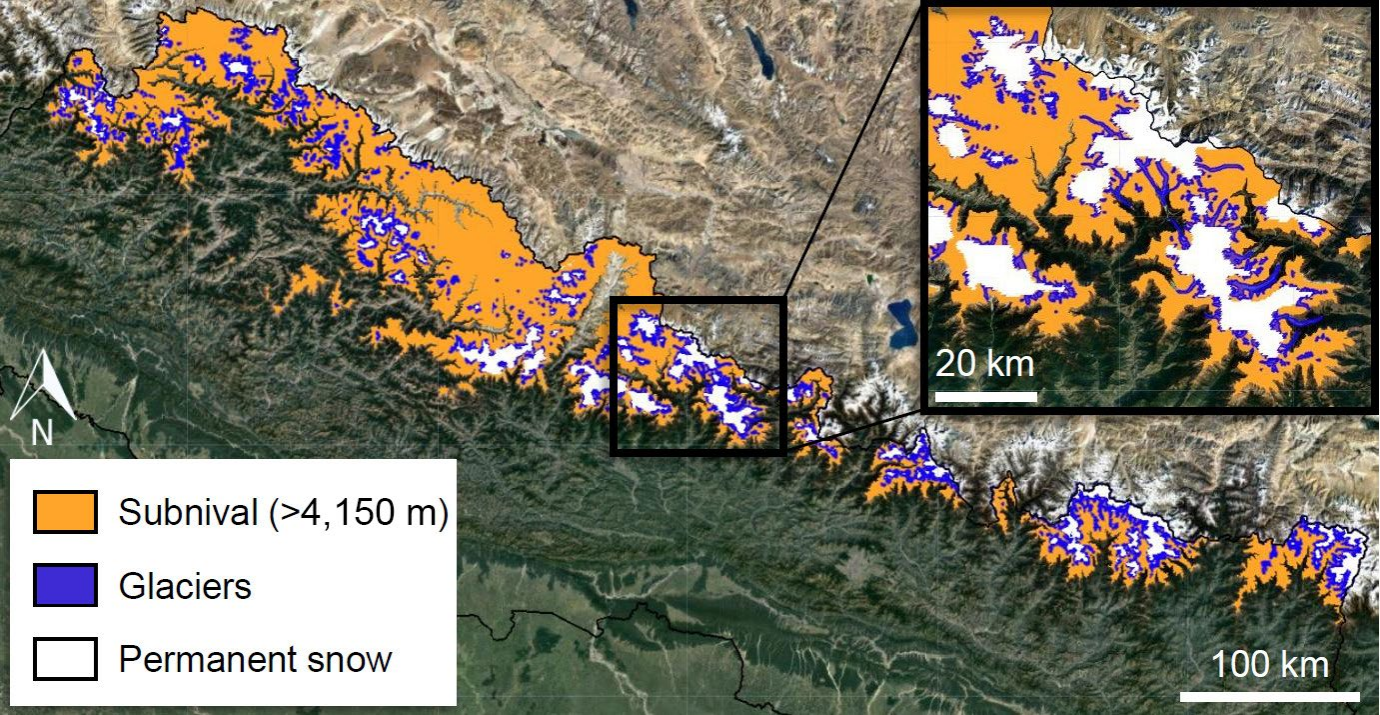
Median snow cover extents over the 2013–2017 period of moderate resolution imaging spectrometer observations for the January–February period (expected maximum extent) and the August–September period (expected minimum extent, permanent snow cover) for Nepal [Colour figure can be viewed at http://www.wileyonlinelibrary.com]

Results showed that an average area of 27,388.4 km^2^ of Nepal lies above 4,150 m a.s.l. (Figure 4; Table 1). According to the RGI (Pfeffer et al., 2014) and MODIS analysis, 4,520 km^2^ of Nepal is covered by permanent ice and snow, resulting in a ratio of seasonal snow to permanent snow/ice cover of 5.1:1. In the P140‐R40/41 region, the ratio is higher at 15.1:1 (Table 1).

**Table 1:** Statistics describing the spatial extent of the subnival zone compared to the permanent snow/ice coverage derived from the RGI and MODIS data. We provide a ratio‐based calculation of the area of the subnival zone to permanent snow/ice

Parameter (km^2^)	P140‐R40/41 region	Nepal
Area of permanent snow cover from MODIS (median of minimal snow cover extent in late summer [August and September]), fraction times area	1,078.1	2,207.1
Glacier cover area (RGI)	1,953.3	3,340.5
Snow area on glaciers	643.6	1,027.6
Total area of permanent snow and ice	2,387.8	4,520.0
Area with elevation >4,150 m a.s.l.	38,513.4	27,388.4
Subnival area without permanent snow and ice	36,125.6	22,868.4
Ratio of subnival area (excluding permanent snow and ice cover) to the permanent snow and ice covered area	15.1:1	5.1:1

Abbreviations: MODIS, moderate resolution imaging spectrometer; RGI, Randolph Glacier Inventory.

### 3.2 Has the spatial extent of subnival vegetation changed and, if so, at what rate and where?

#### 3.2.1 P140‐R40/41 region

**Figure 5:**
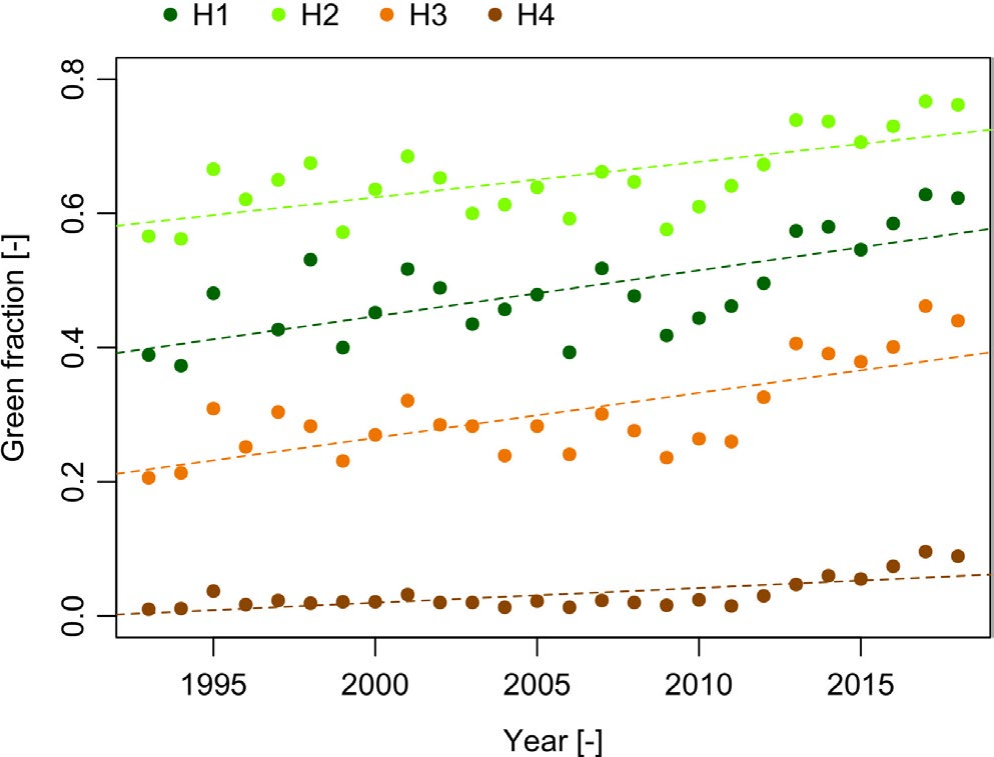
Green pixel fraction time series (1993–2018) for the P140‐R40/41 region centred on P140‐R40/41, based on L5 + L7 + L8 SR collections [Colour figure can be viewed at http://www.wileyonlinelibrary.com]

Figure 5 shows the trends for green fraction against time for the P140‐R40/41 region. For comparison, we also share the results of the trends prior to the Roy et al. (2016) correction of the Landsat data in Table S3. The higher fractional vegetation cover in the H2 height band compared to the H1 height band (Figure 5) is somewhat counterintuitive but closer investigation revealed that this was caused by a local feature within H1 which contained a large meandering riverbed with little vegetation. Table 2 gives the OLS and Mann–Kendall trend test results for these time‐series trends. At all height bands for the P140‐R40/41 region, we found positive, significant (*p* = .05) trends using data corrected according to Roy et al. (2016), with strongest slopes in H1 (OLS slope = 0.0066) and H3 (OLS slope = 0.0067). Trends at the highest elevations (H4) were very weak (OLS slope = 0.002) compared to stronger trends in lower height bands. The non‐parametric Mann–Kendall tests are based on ranked data and produced relatively uniform statistics across all four height bands, describing the strength of relationship between green fraction and time (Kendall's *τ* was between 0.445 and 0.489 for all). The similar values of Kendall's *τ* thus suggest proportional rates of change at different heights.

**Table 2:** Slope, *R* ^2^ and *p* value of OLS regression and Kendall's tau and *p* value of Mann–Kendall trend test of green fractions from 1993 to 2018 for the P140‐R40/41 region

Parameter	Height bands			
	H1	H2	H3	H4
OLS slope	0.0066	0.0053	0.0067	0.0022
OLS *R* ^2^	.475	.433	.515	.478
OLS *p* value	6e‐05	2e‐04	2e‐05	6e‐05
Kendall's *τ*	0.489	0.446	0.445	0.481
Mann–Kendall *p* value	5e‐04	.0015	.0016	7e‐04

Note

Significant results are shaded grey (*p* = .05).

Abbreviation: OLS, ordinary least squares.

#### 3.2.2 Nepal

**Figure 6:**
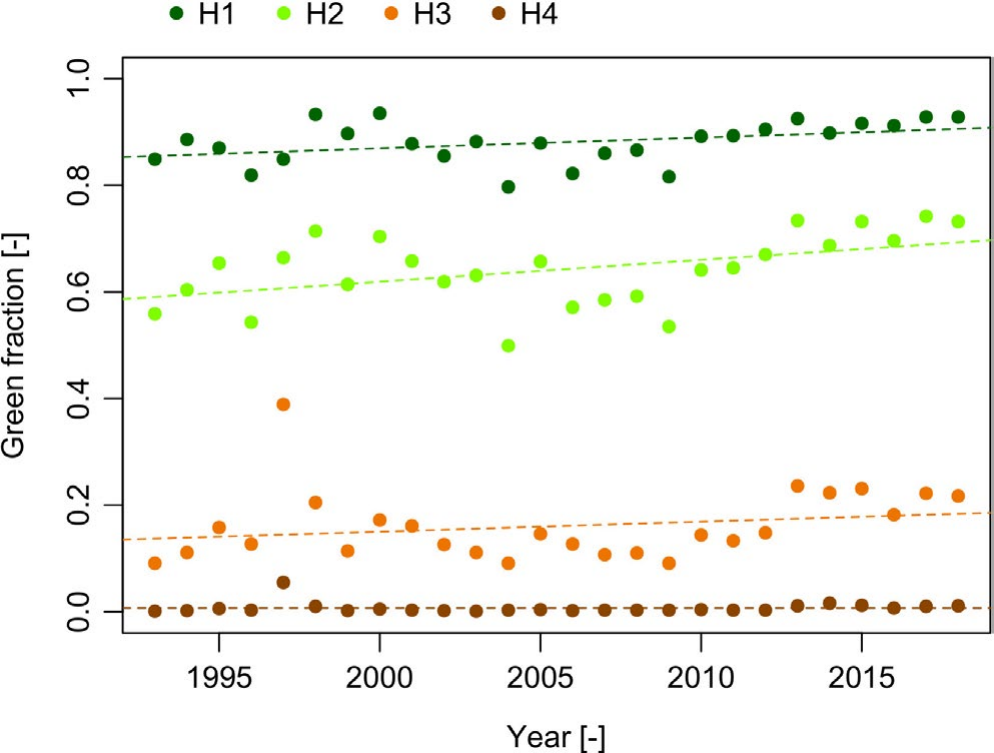
Green pixel fraction time series (1993‐2018) for the area of Nepal based on the L5 + L7 + L8 SR collections [Colour figure can be viewed at http://www.wileyonlinelibrary.com]

We repeated the analysis across the entire spatial extent of Nepal (Figure 6). The OLS slope coefficients were smaller than those found for the P140‐R40/41 region but trends in all four height bands were weakly positive. The only significant, positive trend was found in H1 and H2 using OLS (Table 3). Contrastingly, the Mann–Kendall trend analysis found significant positive trends in H1, H2 and H4. The value for Kendall's *τ* was quite similar for H1, H2 and H4 (0.355, 0.357, 0.344 respectively)—suggesting proportional rates of change for these height regions (Table 3). The outlier shown in 1997 (Figure 6) probably affected OLS analyses for H3 and H4. This outlier was caused by missing data for roughly one‐third of the total area due to missing scenes and cloud/snow cover, and it likely influenced the computation of the green pixel fraction. It only affects data in the higher height bands and is not noticeable in H2 and H1, explained by more snow and cloud at higher elevations.

**Table 3:** Slope, *R* ^2^ and *p* value of OLS regression and Kendall's *τ* and *p* value of Mann–Kendall trend test of green fractions from 1993 to 2018 for Nepal

Parameter	H1	H2	H3	H4
OLS slope	0.002	0.0041	0.0019	−2e‐06
OLS *R* ^2^	.124	.185	.008	−.042
OLS *p* value	.043	.016	.286	.9933
Kendall's *τ*	0.355	0.357	0.242	0.344
Mann–Kendall *p* value	.012	.011	.089	.019

Note

Significant results are shaded grey (*p* = .05).

Abbreviation: OLS, ordinary least squares.

#### 3.2.3 HKH region

**Figure 7:**
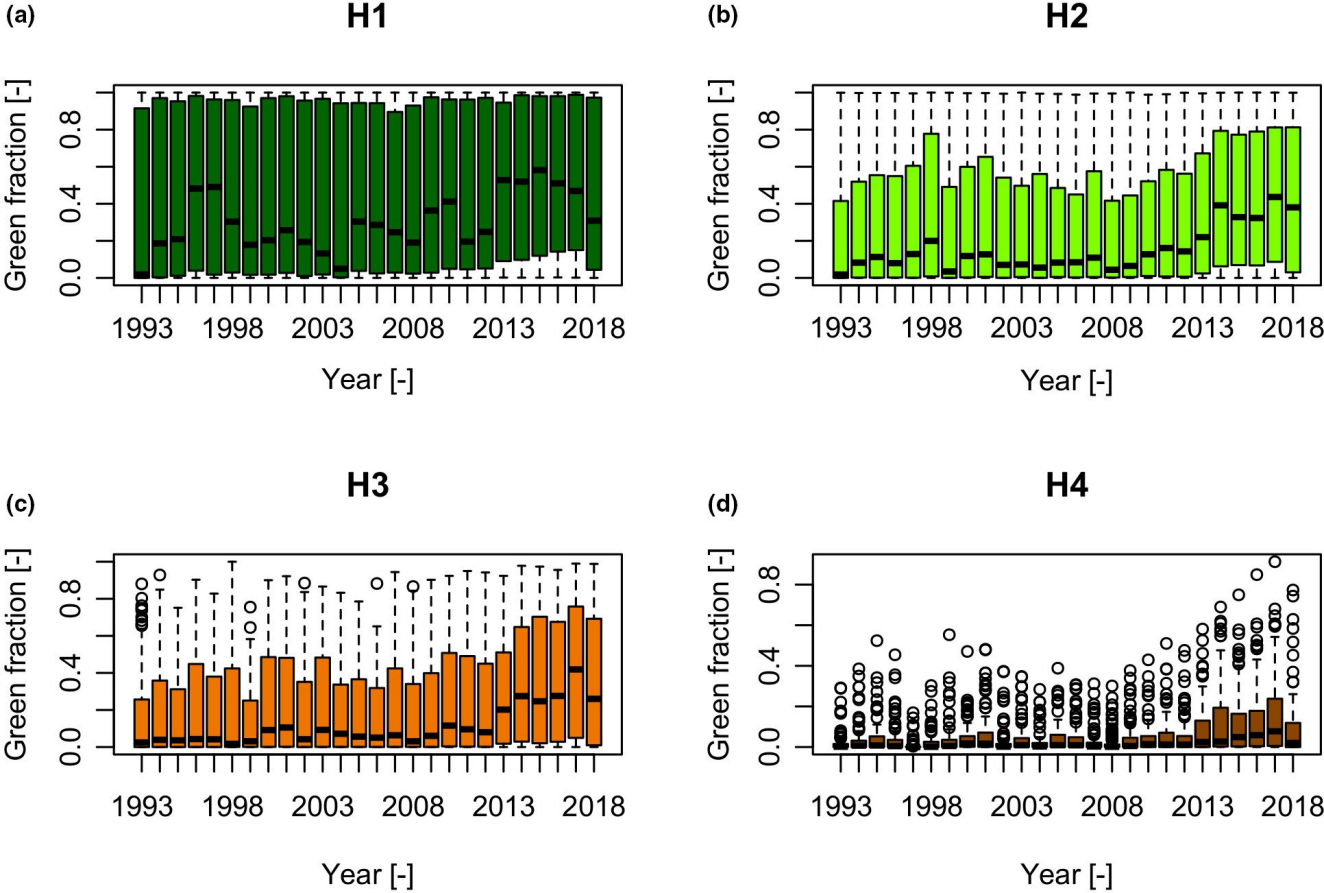
Boxplots of green pixel fraction values for all regions of interest per year derived from the L5 + L7 + L8 SR collections (1993–2018). Plots are separated by height band (a) H1, (b) H2, (c) H3 and (d) H4. The extent of the boxes represent the 25th and 75th percentiles (quartiles), the bold middle line is the 50th percentile (median), the whiskers are the minimum and maximum values which fall within 1.5 times the interquartile range and the circles represent values beyond this range (outliers) [Colour figure can be viewed at http://www.wileyonlinelibrary.com]

For the broader HKH region, the overall pattern of mean green fraction over time show weakly positive time‐series trends (Figure 7; Table 4). As with the more localized investigations, these trends exhibit differences with height with the highest OLS slope coefficients measured in H2 and H3 (0.0061 and 0.008 respectively; Table 4), and the smallest OLS slope coefficients in H4 (0.0035; Table 4). Mann–Kendall trend analysis showed a more nuanced pattern, with similar Kendall's *τ* values for H1, H2 and H4, with the highest Kendall's *τ* in H3 (0.424). Height band H3 having the strongest time‐series trend was thus consistent between OLS and Mann–Kendall trend analysis. These data were collected from 100 ROIs (5 km radius) which necessitated a different type of statistical analysis to determine significance. We evaluated the percentage of ROIs showing trends significantly different from 0 (*p* < .05), and H3 showed the highest percentage of ROIs showing significantly positive trends (76% for both OLS and Mann–Kendall). H1 shows the lowest percentage of significant positive trends (OLS = 56%; Mann–Kendall = 58%). The Mann–Kendall trend analysis found a higher proportion of significant trends compared to OLS in H1 and H2, but less in H4.

**Table 4:** Results for analysis for the entire HKH (1993–2018)

Parameter	Height bands			
	H1	H2	H3	H4
OLS slope	0.0052	0.0061	0.008	0.0035
OLS *R* ^2^	.24	.239	.302	.243
OLS *N* sig.	56%	57%	76%	63%
Kendall's *τ*	0.335	0.354	0.424	0.35
Mann–Kendall *N* sig.	58%	67%	76%	60%

Note

OLS parameters and Mann–Kendall test statistics are given for green pixel fraction over time per height band calculated using 100 circular ROIs with 5 km radius over the entire HKH, as well as the percentage of ROIs showing trends significantly different from 0 (*p* < .05).

Abbreviations: HKH, Hindu Kush Himalaya; OLS, ordinary least squares; ROI, region of interest.

To provide an independent test of time‐series trends where sensor configuration was constant, for the HKH‐wide data set, we also calculated the time‐series trend using SR data from Landsat 5 and 7 TM and ETM+ sensors only (i.e. excluding L8 OLI data). Results are shared in the Supplementary Information (Figure S5; Table S4). We found similar positive time‐series trends to the full analysis shown in Figure 7, although these trends were weaker when L8 data were excluded (Figure S5) as compared to when they were included (Figure 7). OLS slope coefficients were positive, and Kendall's *τ* similarly showed the highest value in H3 (Table S4), corroborating results from the full analysis. Excluding L8 data resulted in fewer ROIs showing positive trends significantly different from 0 (*p* < .05) compared to the full analysis, but all height bands still contained ROIs with positive trends. As in the full analysis, H3 had the highest number of ROIs showing significant positive trends (45%; Table S4).

### 3.3 Geographic effects: Slope and aspect

**Figure 8:**
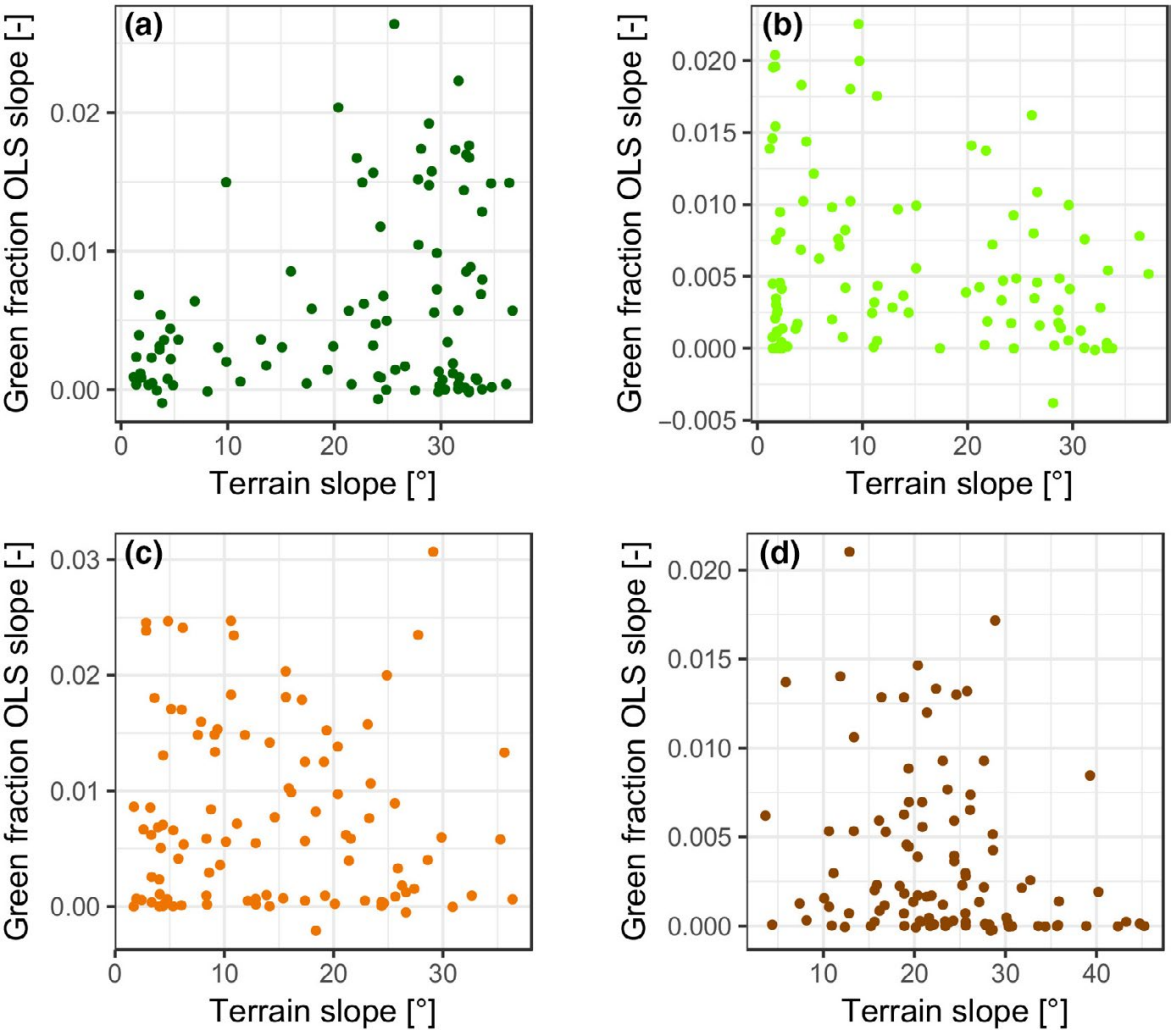
Green fraction (1993–2018) plotted against the median terrain slope value per ROI for the L5 + L7 + L8 collection over the HKH‐wide extent within four height bands (a) H1; (b) H2; (c) H3; (d) H4 [Colour figure can be viewed at http://www.wileyonlinelibrary.com]

A final analysis determined whether there were differences in the slope or direction of the time‐series vegetation response given different underlying geographic conditions—specifically slope and aspect. This analysis was performed with the HKH‐wide data set. First, slope angle was queried within the four height bands (Figure 8). Whilst there is a lot of scatter, the slope of green fraction trends is more positive for steeper terrain slopes in H1 (Figure 8a) whilst for H2–H4, the patterns with slope trend slightly more towards negative behaviour as elevation increases (Figure 8a through d). Analysis with aspect showed no visually discernible or statistically meaningful patterns, so we have not included a plot.

### 3.4 Snow fractional cover change over time

The time series of snow fractional cover showed no significant trend when analysed with the Mann–Kendall rank test for the P140/R40‐41 region and Nepal extent (see Figure S3). For the 100 ROIs distributed HKH‐wide, we found an increasing number of ROIs that showed weak, yet significant, negative trends in snow fraction, with increasing height. In H1, 7% of ROIs showed a significant negative snow fraction trend over the time series, increasing to 23% in H4 (see Figure S4; Table S2).

### 3.5 Validation

**Figure 9:**
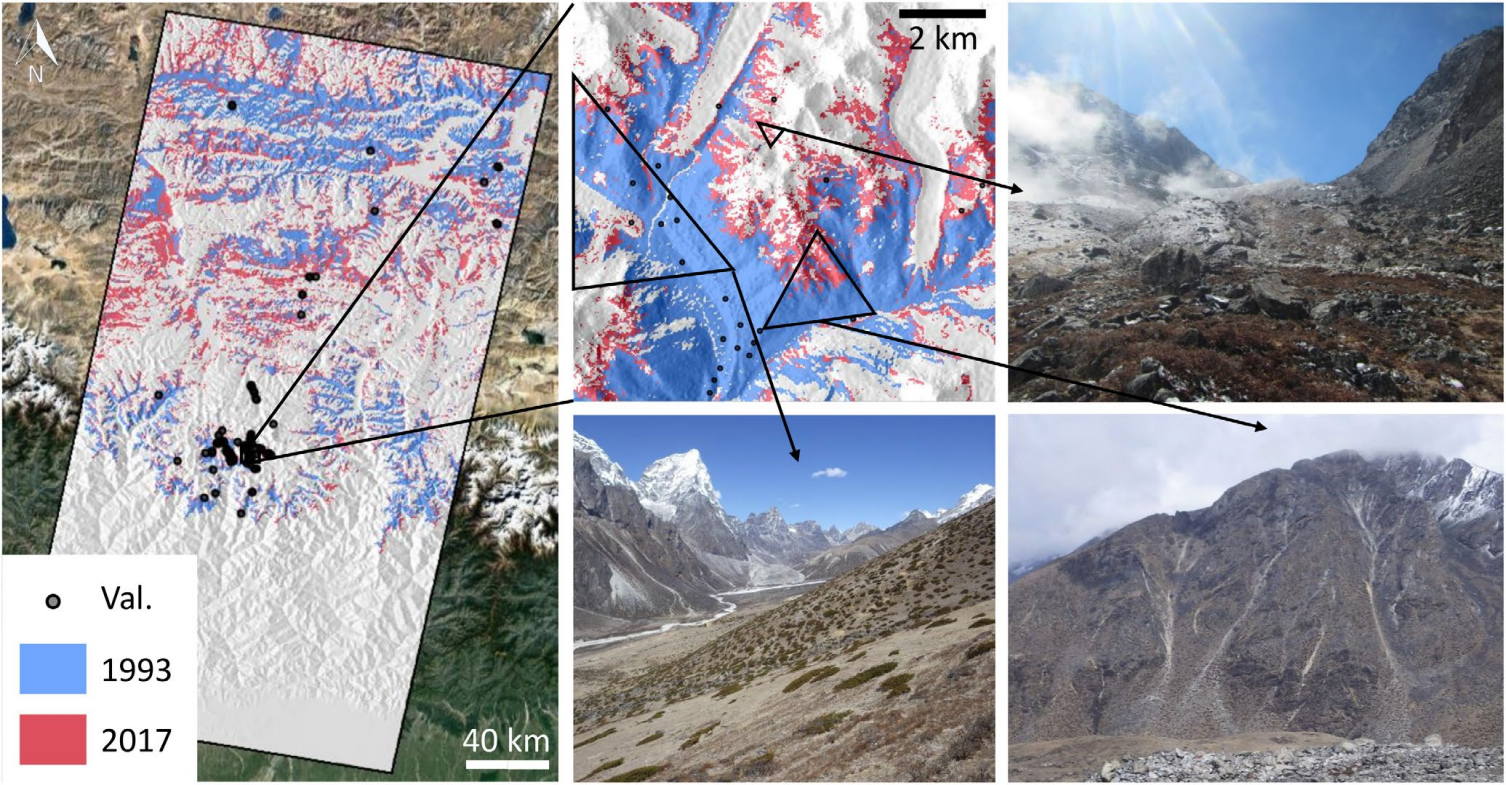
Validation of the vegetation product from 2017 within the P140/R40‐41 region (red pixels). Field photographs shown were captured in the Khumbu region of Nepal during 2017 (authors' own). All photographs show the presence of vegetation in pixels that were also labelled as vegetated according to the Google Earth Engine (GEE) analysis. Validation points highlighted are the locations of the 98 points where our own photos, plus those from Google Streetview and Photosphere were obtained for validation. Underlying vegetation extent maps represent the median Landsat composites including all masks for October and November 2017 (red), and for 1993 (blue) which was the earliest year considered in the GEE time‐series analysis [Colour figure can be viewed at http://www.wileyonlinelibrary.com]

We evaluated the utility of photographic data for providing presence/absence information about vegetation in the subnival HKH. We report that photographs available through Google's ‘Streetview’ and ‘Photosphere’ products, and our own field photographs, contained useful ecological information about the presence or absence of broad vegetation groups (grasses, mosses, shrubs and bare ground). Figure 9 shows a small selection of our own photographs, all captured in 2017 from the Khumbu region of Nepal. These were used alongside Google images to validate the satellite‐derived vegetation product from 2017 for the P140‐R40/41 region. Red pixels in Figure 9 show the vegetated extent in 2017, whilst blue pixels show the same for 1993, which was the earliest year in the time‐series analysis considered previously. The extent of blue pixels (1993) is considerably smaller than that of red pixels (2017), providing visual evidence for expansion trends reported for P140/R40‐41 earlier.

A total of 194 individual observations were gathered from both Google and our own photographic collections. From the collected observations, any based on images acquired prior to 2014 were excluded (4%), with the remaining observations considered recent enough for the validation of the 2017 NDVI thresholded product (red pixels in Figure 9). Remaining observations were filtered spatially using a distance threshold of 100 m to remove duplicates, and points coinciding with snow or north facing slopes were also excluded. This resulted in 98 unique point observations for validation (dots shown in Figure 9), containing presence/absence vegetation information at each point. Validation of the satellite product for the P140‐R40/41 region above an elevation of 4,150 m a.s.l. revealed an overall accuracy of 79.6% (Table 5). The errors of omission and commission for the two classes are provided in Table 6. In the pixels falsely classified as unvegetated (*n* = 13), 53.8% contained shrubs, 30.8% grasses and 38.5% mosses.

**Table 5:** Confusion matrix for the reference and classified vegetated and unvegetated pixels

		Reference data set		Sum classified
		Vegetated	Unvegetated	
Classified data set	Vegetated	65	7	72
	Unvegetated	13	13	26
Totals		78	20	98
Overall accuracy (65 + 13/98)				79.6%

Note

Correctly classified are shaded grey.

**Table 6:** Omission and commission error percentages for the vegetated and unvegetated pixels, including the number of reference observations for the two classes

	Vegetated	Unvegetated
Reference *n*	78	20
Error of omission	16.7%	35.0%
Error of commission	9.7%	50.0%

## 4 DISCUSSION

A wealth of scientific work has highlighted that the HKH region is changing in response to climate (Keenan & Riley, 2018; Pachauri et al., 2014; Schickhoff, 2011), and whilst great research efforts have been invested in high altitude cryospheric and climatological work (Bajracharya et al., 2015; Gardner et al., 2013; Gautam, Hsu, Lau, Tsay, & Kafatos, 2009; Sano, Ramesh, Sheshshayee, & Sukumar, 2012; Shrestha, Wake, Mayewski, & Dibb, 1999), studies that address high altitude ecological responses are comparatively lacking (Gaire et al., 2014). Our work has advanced ecological understanding of the poorly studied HKH subnival system by being the first to measure the extent of changes in the fractional cover of subnival vegetation in the HKH (4,150–6,000 m a.s.l.). Specifically, we have shown that significant changes have occurred in vegetation distribution measured through ‘green fraction’ analyses of Landsat 5, 7 and 8 data since 1993. Our analyses have incorporated a robust cross‐sensor calibration to correct for sensor differences between the Landsat 7 ETM+ sensor and the Landsat 8 OLI, which have different radiometric sensitivities. Taking a fully precautionary approach and excluding data from L8 OLI, across the HKH extent, we still found significant positive trends in vegetation fractional cover over time, although those trends were weaker without, than with L8 data included in the time series (Figure S5; Table S4). We have shown that the satellite‐derived green fraction product can be validated using freely available photographic data with good accuracy (79.6%; Tables 5 and 6).

### 4.1 What is the *extent* of the subnival zone?

Subnival habitat in the Nepalese and P140/R40‐41 regions, which are microcosms of the broader HKH system, covers between five and 15 times the area of permanent glaciers and snow (Table 1). These ratios indicate the extent of seasonal snow cover that could be impacted by future changes in vegetation cover. There is a much larger proportion of the high altitude Qinghai–Tibetan plateau (QTP) within the region of the P140‐R40/41 scene (15.1:1) than in Nepal, which exhibits a smaller ratio (5.1:1); and the QTP exhibits greater seasonal snow storage than the steep mountainous areas of Nepal. Across the HKH region generally, it is reasonable to assume that subnival snow to permanent snow/ice spatial coverage will likely vary between the Nepalese and P140‐R40/41 regional ratios: areas on the plateau exhibiting higher ratios whilst areas with steeper topography will have lower ratios. Of importance, beyond areal estimates are snow depth and snow water equivalence which impact hydrological regimes: we could not evaluate those with the satellite data used here, but further studies should seek to advance such understanding. Regardless of geographical location, these results indicate that the subnival zone should be recognized for its likely role in influencing non‐base flow water supplies, with impacts on downstream communities. The lack of science questioning the role of subnival vegetation in HKH ecohydrology, with impacts scaling from headwater catchments to the spatial extent of the ‘third pole’ (http://www.icimod.org/?q=3487) is a gap that requires urgent scientific attention.

Whilst rapidly changing Arctic ecosystems have received considerable investment in ecological research, the same cannot be said for Himalayan ecosystems (Gurung & Bajracharya, 2012), despite the much larger number of people reliant on mountain water supplies from the HKH than in the Arctic (Immerzeel et al., 2010; Pritchard, 2019). Yet, there is a potential nexus of impacts (particularly hydrological impacts) that could arise from vegetation expansion at high elevation. Future studies need to establish vegetation impacts on, and feedbacks to HKH: snowpack (Brandt et al., 2013), permafrost (LaMadrid & Kelman, 2012), phenology (Smith, Sconiers, Spasojevic, Ashton, & Suding, 2012), surface temperatures (Myers‐Smith et al., 2011) and snow melt rates (Blok et al., 2011; Pomeroy et al., 2006; Wookey et al., 2009), since Arctic work has shown that vegetation changes can impact these processes profoundly. Readers should note that Arctic work focuses strongly on changes in species/functional composition rather than the conversion of bare ground to vegetated as explored here, but we argue that the latter might be expected to deliver more profound hydrological impacts. Importantly, work emerging from Tibet shows a contrasting picture to Arctic plant/snow feedbacks, with evapotranspiration‐driven cooling outweighing albedo‐driven warming in shrub‐dominated systems (Shen et al., 2015). Future in situ work that uncovers the functional relationships between Himalayan subnival plants and hydrology would provide timely answers to these questions, whilst modelling approaches which test the eco‐hydrological impacts of vegetation expansion will deliver new insights in the HKH (e.g. following work such as Rasouli, Pomeroy, and Whitfield (2019)). We also suggest that future remote sensing work that delivers spatial information about plant traits (e.g. grass/shrub land cover mapping) over large areas could deliver new understanding about the wider mechanisms and impacts of vegetation expansion.

### 4.2 Has the spatial extent of subnival vegetation changed and, if so, at what rate and where?

Using NDVI derived from Landsat data records since 1993 to distinguish between vegetated and non‐vegetated pixels, we showed an increase in green fractional cover over time. Exploring these trends over three spatial extents and within four elevation bands, we found that at all scales and elevations, positive trends in vegetation were found between 1993 and 2018, but these were not always statistically significant. In summary:

1. An area centred on Mount Everest (P140/R40‐41) showed significant positive trends in all four height bands (OLS and Kendall's *τ*; Table 2);
2. At the national scale of Nepal vegetation trends in H1 and H2 (OLS and Kendall's *τ*) and H4 (Kendall's *τ* only) showed significant positive trends (Table 3);
3. Across the HKH extent, using 100 randomly distributed ROIs to sample time series all height bands between 56% (H1; OLS) and 76% (H3; OLS and Kendall's *τ*) of ROIs showed positive trends significantly different from 0 (*p* < .05; Table 4).

From this work, we conclude that subnival vegetation expansion is occurring HKH‐wide, although there are clearly geographic differences in the strength and significance of that trend. This is to be expected given the topographic and climatic diversity of the HKH (Bookhagen & Burbank, 2006). The observed trends are consistent with modelling work showing a decline in temperature‐limited areas resulting from warming of the Earth's cold regions (Keenan & Riley, 2018), and these results also corroborate the work of Gonzalez, Neilson, Lenihan, and Drapek (2010) who declare Himalayan ecosystems as being highly vulnerable to climate‐induced vegetation shifts.

Our analysis revealed variations in rates of change with elevation. For the height band H2 (4,500–5,000 m), trends were positive and significant for P140‐R40/41 and for Nepal with between 57% (OLS) and 67% (Kendall's *τ*) of trends across the entire HKH showing significant positive trends. However, on the HKH scale, a higher percentage of ROIs with significant trends were found in H3 (76% of ROIs; OLS and Kendall's *τ*) with a stronger association with lower terrain slopes (Figure 8c), likely indicating an increase of green pixels on the extensive QTP to the north of Nepal. Combined, the H2 and H3 height bands cover most of the QTP area for which models predict an increase in shrub vegetation at the cost of alpine meadow, steppe and desert (Zhao, Wu, Yin, & Yin, 2011). It is possible that the observed positive trends are linked to this regime shift, also reported by Brandt et al. (2013); however, it is unclear whether the NDVI threshold‐based analysis is sensitive to a change to a different vegetation type.

Trends were weakest in the highest elevation band, H4 (5,500–6,000 m a.s.l.) across all three scales of our analysis. We hypothesize that this was due to temperature limitation and overall steeper slopes which are less suitable for vegetation colonization due to regular disturbance from peri‐ or paraglacial activity (e.g. rockfalls and landslides; Ives & Messerli, 1989). Where increases were found to occur in H4, these were associated with gentler slopes (Figure 8d), as these may be less susceptible to erosion and plant colonization. The opposite was found to be true for areas at lower elevations (e.g. H1; Figure 8a)—with higher green fraction model slopes on steeper terrain slopes. We suggest that this is a function of lower lying areas having high vegetation cover at the start of the time series, and thus, vegetation expansion is more likely to occur on steeper terrain at lower elevations.

The analysis of fractional snow cover through time showed no underlying trend at either the P140/R40‐41 or Nepalese scale, but at the HKH extent, we found increasing numbers of ROIs showing significant weak negative trends as elevation increased to a maximum of 23% in H4 (Figures S3 and S4; Table S2). Under climate change, the area covered by snow could be impacted, with some evidence showing snow line altitude increases over time in the HKH (Pandey, Kulkarni, & Venkataraman, 2013). Our results suggest that there is some evidence for increased snow line altitudes, and this could be a mechanism through which significant vegetation expansion can occur (i.e. through reduction in temperature limitation; Keenan & Riley, 2018 and increased availability of bare ground for propagation). Further exploration of the spatiotemporal patterns at the snow–vegetation interface is warranted in this regard.

### 4.3 Critical evaluation of Landsat time‐series data

The linear model‐based Roy et al. (2016) correction is a simple approach to resolve the L7–L8 sensor transition and has been used by others in similar settings (Fassnacht et al., 2019). As shown in Table S3, applying the Roy et al. (2016) model to the data for the P140‐L40/41 ROI caused OLS slopes and *R* ^2^ values to be slightly reduced but did not impact the significance of relationships determined with OLS or Kendall's *τ*. When using long time‐series data sets from different sensors, there are other issues to consider. For Landsat, these include failure of the Scan Line Corrector mechanism in L7, during 2003 (Markham, Storey, Williams, & Irons, 2004), resulting in the loss of approximately 22% of each scene (USGS, 2017). We have also considered the limitations of the NDVI product which can be susceptible to bare soil‐induced background noise, and the added uncertainty caused by the slightly different atmospheric correction approaches in Landsat 8 versus Landsat 7 and their impact on unvegetated pixels (which we found to show no significant time‐series trend—see Figures S1 and S2; Table S1). Topographic effects in the HKH may have added further uncertainty, but we mitigated this by excluding the highly shadowed north‐facing slopes. Whilst NDVI is a relatively simple index, new indices developed to alleviate issues with soil background or atmospheric effects have been unable to improve on NDVI in shrub‐dominated landscapes (Gaitán et al., 2013). Alongside our own study, there are plentiful examples of ecological studies using Landsat‐scale NDVI products in shrub/grass‐dominated or alpine systems (Carlson et al., 2017; Marston et al., 2017; Riihimäki, Heiskanen, & Luoto, 2017), so resultantly, we argue that our NDVI thresholding method is suitable for deriving a reliable binary presence/absence vegetation discrimination for the subnival zone.

We suggest caution, as do others (Holden & Woodcock, 2016; Roy et al., 2016), in the application of Landsat time‐series data to similar questions, given the various changes in sensor configuration that have been implemented through the Landsat generations. Our work has shown that when taking a precautionary approach and excluding L8 OLI sensor data from the HKH‐wide analysis completely, time‐series trends in L5 and L7 (TM and ETM+ sensor data) were still evident: most markedly in H3, but within fewer ROIs and producing trends with weaker OLS slopes. The weaker slopes/fewer significant ROIs could result from various factors, notably sensor issues with L7 as it approaches the end of its mission life, L8 OLI bandset differences when compared to L7 ETM+ and L5 TM (although these should be accounted for by our use of Roy et al., 2016's coefficients) or the reduction in sample size when L8 data were removed. At the time of writing, the Roy et al. (2016) correction is the state‐of‐the‐art method for handling the L7–L8 sensor transition and it was designed for use with applications such as this. Similar future work should take care to account for sensor differences (as we have done here), and to correct for them appropriately using state‐of‐the‐art approaches. Furthermore, use of a calibrated SR product is not assurance that such issues have been taken care of fully by data providers—indeed, validation of products from operational Landsat missions (e.g. L7 and L8) by the Landsat science team is an ongoing process (see1
https://www.usgs.gov/land-resources/nli/landsat/landsat-science-team-meetings
).

### 4.4 Validation

We have demonstrated the scientific value of open‐source photographic data for validation purposes in remote areas where in situ validation data do not exist. Unlike scientific photographic methods for in situ vegetation characterization (e.g. Phenocam network; Brown et al., 2016; Sonnentag et al., 2012), the utility of Google Streetview and Photosphere images is limited to the identification of the broad type of vegetation cover, but this basic ecological information remains highly useful. One issue we found is that since the availability of such imagery is largely from lower elevations, there were few examples of photographs containing no vegetation, so it was quite challenging to obtain a large sample size with which to evaluate the accuracy of Landsat products for mapping bare ground. Also, the positional uncertainty of these geotagged photos is not known, which is why we used a 100 m buffer to eliminate potential replicate observations. Validating the 2017 NDVI thresholded product (which contained observations from both L7 ETM+ and L8 OLI), we found that 79.6% of pixels were accurately classified, highlighting at least for the recent data, some of the uncertainties in the method. We assert that online repositories of photographs contain a rich source of archival data for answering basic ecological questions or performing validation in hard‐to‐reach areas, and yet they remain almost completely untapped as a source of data for poorly instrumented regions of the world.

### 4.5 Google Earth Engine

This work evidences the power of using cloud‐based computation of remote sensing analyses. To undertake the analyses described in this manuscript using traditional remote sensing workflows would have necessitated the use of a very powerful computer and required the downloading of thousands of individual Landsat scenes. For ecologists interested in studying large‐scale ecosystem processes over extensive spatial areas and over multi‐decadal timescales, and for those working in places without access to supercomputing facilities, the capabilities offered by GEE are unparalleled. We used Earth Engine v0.1.201 and the Javascript Application Programme Interface for all processing. We share our GEE code openly in the spirit of science as a transparent endeavour. A link to the code is provided in the Supplementary Information.

## 5 SUMMARY

The HKH are Asia's water towers (Immerzeel et al., 2010). It is accepted that water supplies in the HKH are threatened as a result of climatic shifts (Bolch et al., 2019; Pritchard, 2019; Shannon et al., 2019), and that plants and the hydrological cycle are coupled (Fatichi et al., 2016). Despite this, science has not so far questioned the extent to which high altitude ecological shifts or transitional processes across the HKH will impact water or carbon cycle processes. A first step towards understanding the role of vegetation in HKH ecohydrology is to measure the extent of the ecosystem relative to snow and ice cover, and to determine whether the ecosystem has changed over time. Our results show vegetation expansion is occurring at high altitudes (>4,150 m a.s.l.) across the HKH and that subnival systems cover between five and 15 times the area of permanent ice and snow. Supported by this evidence, we argue that subnival ecological systems play an important role in HKH hydrology and their role will increase as snowlines ascend and glaciers melt. There is an urgent need for new science to uncover the status, role and fate of high altitude ecosystems in the unique setting of the HKH for modulating seasonal non‐base flow water supplies and cycling carbon. Further scientific work in this inaccessible region will undoubtedly rely heavily on the Earth observation data analysis, as we have performed here, but there is an accompanying, pressing need for new in situ studies. Overall, we need an improved functional understanding of the state and fate of subnival ecosystems and their ecohydrology and such data would also prove valuable for validating satellite‐based analyses.

## AUTHOR CONTRIBUTIONS

KA conceived the original idea for the work, following fieldwork with DJ in the Khumbu region of Nepal. SB and AC undertook individual research projects to address the central research questions over limited spatial areas in the HKH, using traditional RS workflows. KA and DF collaboratively defined the experimental design for satellite data analysis in GEE. DF carried out the coding within GEE and led the practical implementation of the work. KA led the writing of the manuscript, assisted primarily by DF. DJ provided photographic data and worked with DF on validation, and provided feedback on manuscript drafts. RL undertook practical work within GEE to test Landsat time‐series continuity, and was supported by DF and KA whilst visiting the United Kingdom during a research exchange programme. AC and SB assisted with early literature searches and provided input during writing and editing stages of the manuscript.
