# Supplementary material for: Vegetation expansion in the subnival Hindu Kush Himalaya
Source: Glob Chang Biol. 2020 Jan 9;26(3):1608–25. doi: 10.1111/gcb.14919 (PMC7078945; doi:10.1111/gcb.14919)
Supplement: Supplementary file 1 [file GCB-26-1608-s001.docx]

**Vegetation expansion in the subnival Hindu Kush Himalaya**

**Supplementary information**

**
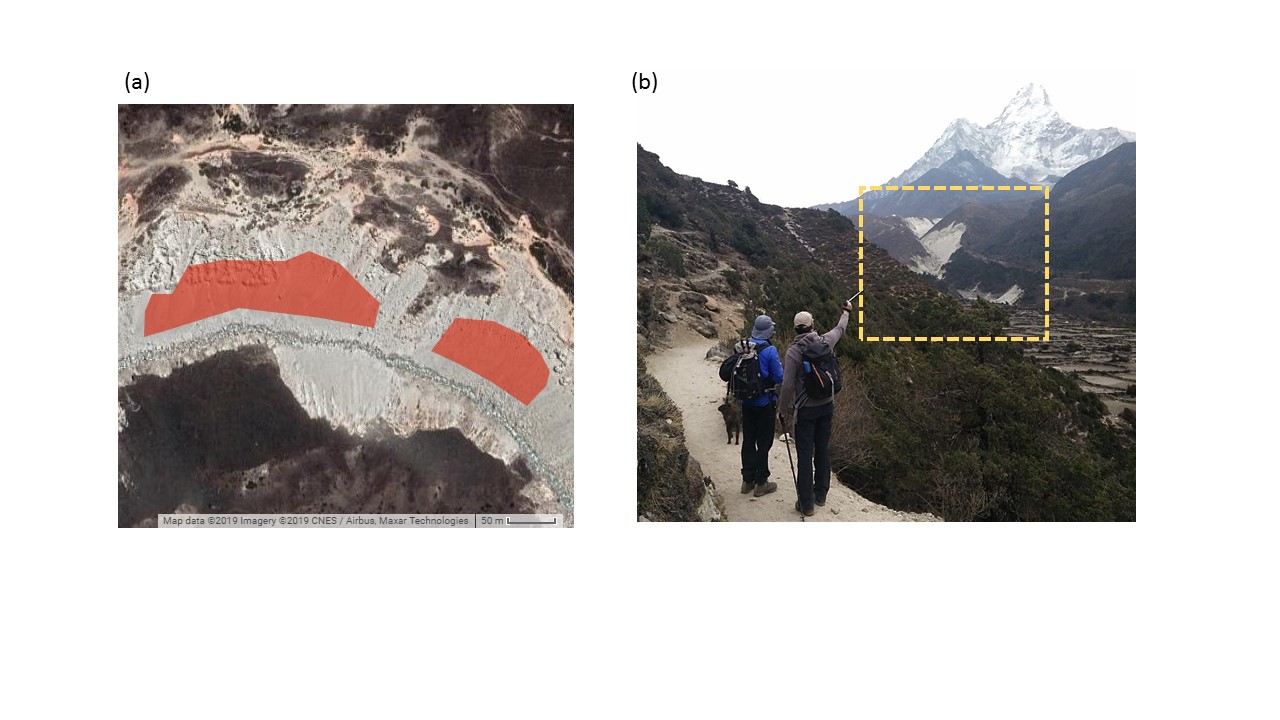
**

**Figure S1: Images of the unvegetated Glacial Lake Outburst Flood (GLOF) scar near Panboche, Nepal. (a) The satellite viewpoint showing the location of two regions of interest from which SR data were sampled; (b) The feature *in situ*, evidencing the lack of vegetation, and imaged by the author (Anderson) from the path near Panboche in April 2017; the peak in the background is Ama Dablam (6812 m.a.s.l).**


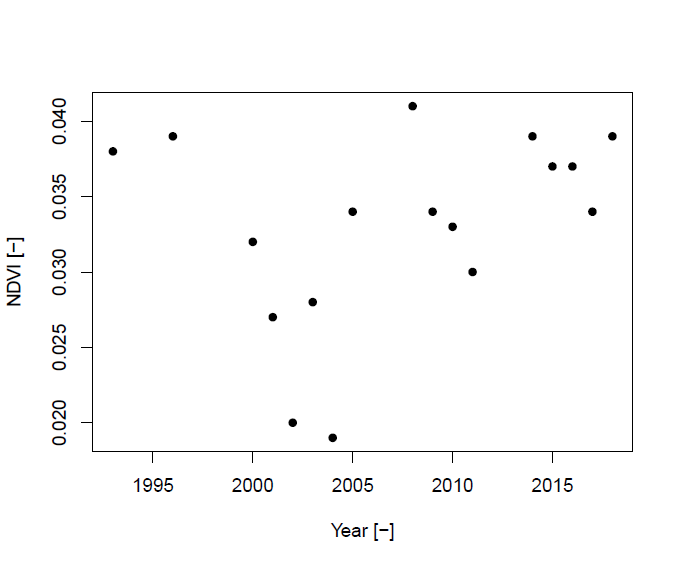


**Figure S2: Landsat surface-reflectance time-series in NDVI from the GLOF scar near Panboche, Nepal. Data here were corrected using the Roy *et al.* (2016) method.**

**Table S1: Ordinary Least Squares (OLS) regression coefficients and significance, and Mann-Kendall test coefficients and significance for the time-series shown in SI Figure 2, indicating the absence of a statistically significant time series trend in this feature.**

| OLS Slope | 0.0002 |
| --- | --- |
| OLS R^2^ | 0.0161 |
| OLS p-value | 0.279 |
| Kendall’s τ | 0.204 |
| Mann-Kendall  p-value | 0.281 |


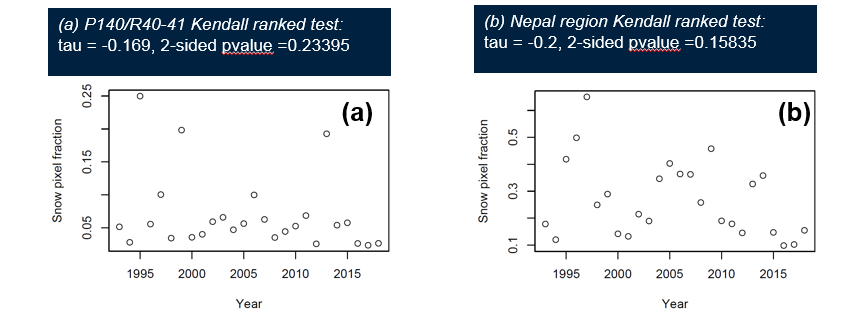


**Figure S3: Snow cover fractional product time-series analysis for the 4150 – 6000 m subnival zone area, based on Landsat QA flags, for (a) P140/R40-41 and (b) Nepal, which showed no significant trends through time.**


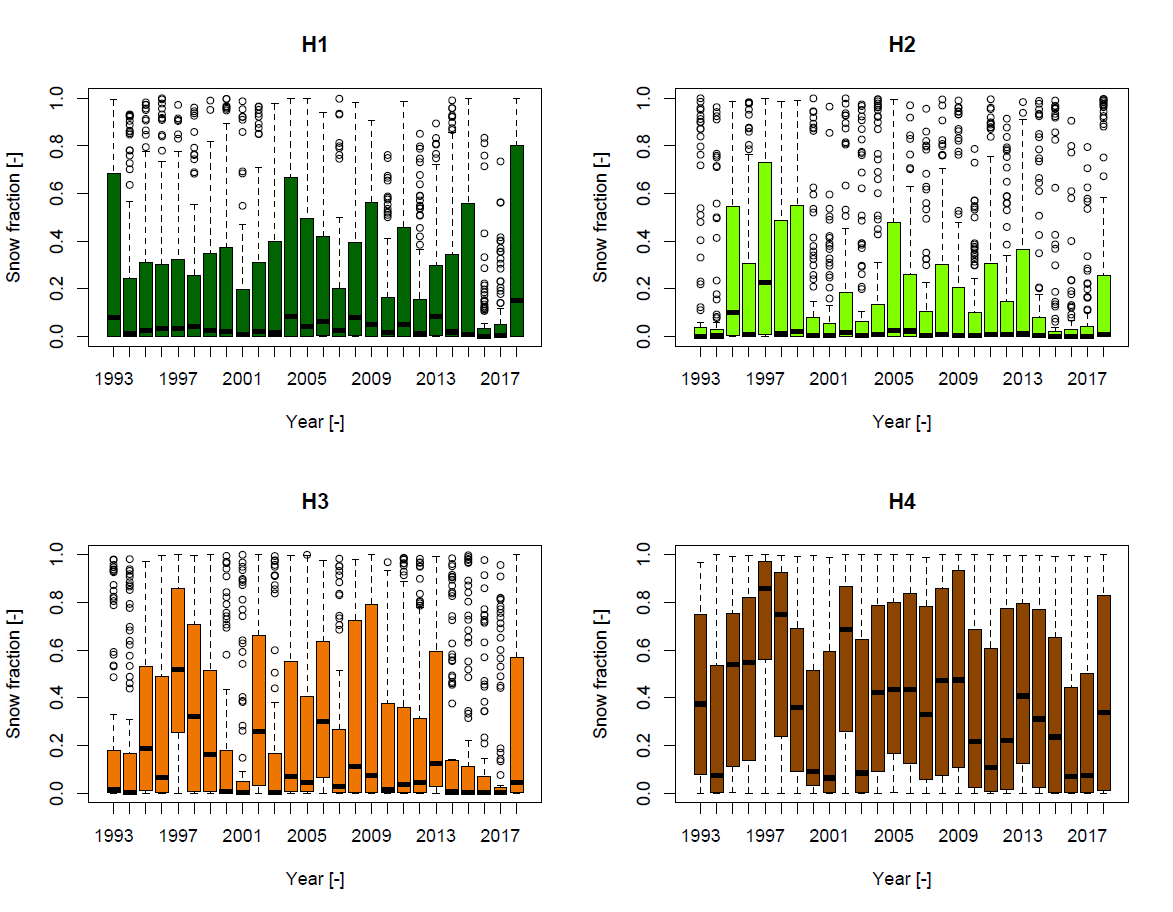


**Figure S4: Snow cover fractional product time-series based on Landsat QA flags for the n=100 regions of interest distributed HKH-wide, within different height bands. SI Table 2 shows the statistical analysis of these trends.**

**Table S2: Mann-Kendall test statistics for snow fraction over time per height band calculated using 100 circular ROIs with 5 km radius over the entire HKH, as well as the percentage of ROIs showing trends significantly different from 0 (p-val <0.05).**

| **Height band** | **Kendall’s τ means** | **N significant (MK p-value < 0.05)** |
| --- | --- | --- |
| H1 | -0.0268 | 7 |
| H2 | -0.0812 | 16 |
| H3 | -0.0758 | 20 |
| H4 | -0.1040 | 23 |

**Table S3: results of statistical trend analysis for OLS and Kendall’s τ prior to, and following application of the (Roy *et al.*, 2016) linear method for correcting L7 ETM+ and L8 OLI data for the P140/R40-41 region of interest. Grey shaded cells are significant results (P>0.05).**

| **L5+L7+L8 (Without Roy et al (2016) correction applied)** | | | | |
| --- | --- | --- | --- | --- |
| **Parameter** | **H1** | **H2** | **H3** | **H4** |
| OLS Slope | 0.0068 | 0.0055 | 0.007 | 0.0024 |
| OLS R^2^ | 0.483 | 0.442 | 0.519 | 0.482 |
| OLS p-value | 5e-05 | 1e-04 | 2e-05 | 5e-05 |
| Kendall’s τ | 0.489 | 0.446 | 0.445 | 0.481 |
| Mann-Kendall  p-value | 5e-04 | 0.0015 | 0.0016 | 7e-04 |
| **L5+L7+L8 (With Roy et al (2016) correction applied)** | | | | |
| **Parameter** | **H1** | **H2** | **H3** | **H4** |
| OLS Slope | 0.0066 | 0.0053 | 0.0067 | 0.0022 |
| OLS R^2^ | 0.475 | 0.433 | 0.515 | 0.478 |
| OLS p-value | 6e-05 | 2e-04 | 2e-05 | 6e-05 |
| Kendall’s τ | 0.489 | 0.446 | 0.445 | 0.481 |
| Mann-Kendall  p-value | 5e-04 | 0.0015 | 0.0016 | 7e-04 |


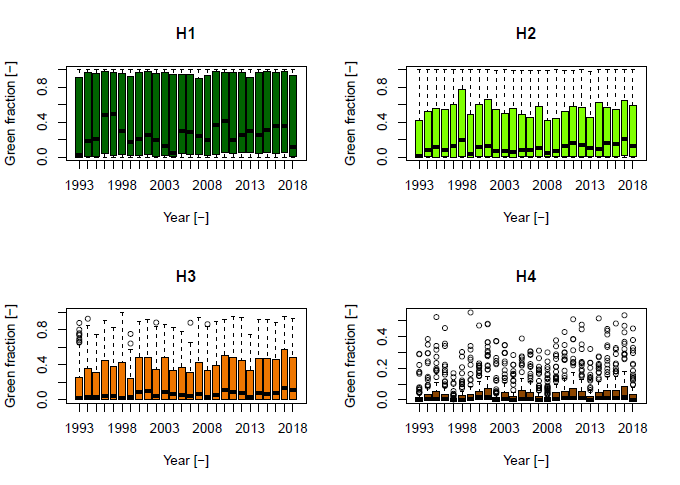


**Figure S5: Boxplots of green pixel fraction values for all ROIs per year derived from the L5+L7 (i.e. excluding L8) SR collections (1993-2018). Plots are separated by height band (a) H1, (b) H2, (c) H3 and (d) H4. The extent of the boxes represent the 25^th^ and 75^th^ percentiles (quartiles), the bold middle line is the 50^th^ percentile (median), the whiskers are the minimum and maximum values which fall within 1.5 times the interquartile range and the circles represent values beyond this range (outliers).**

**SI Table 4: Results for trend analysis for the entire HKH (1993-2018) when L8 data were excluded from the analysis (see SI Figure 5). OLS parameters and** **Mann-Kendall test statistics are given for green pixel fraction over time per height band calculated using 100 circular ROIs with 5 km radius over the entire HKH, as well as the percentage of ROIs showing trends significantly different from 0 (p-val <0.05)**

| **Parameter** | **Height bands** | | | |
| --- | --- | --- | --- | --- |
|  | **H1** | **H2** | **H3** | **H4** |
| OLS Slope L5+L7 | 0.0023 | 0.0019 | 0.0033 | 0.0006 |
| OLS R^2^ L5+L7 | 0.113 | 0.09 | 0.136 | 0.066 |
| OLS N sig. L5+L7 | 22% | 17% | 37% | 4% |
| Kendall’s τ L5+L7 | 0.177 | 0.171 | 0.238 | 0.186 |
| Mann-Kendall N sig. L5+L7 | 24% | 25% | 45% | 13% |

**Google Earth Engine Code**

We share our code for the Google Earth Engine analyses through a GitHub repository which is publicly available at <https://github.com/karenanderson-exeter/HKH_veg_expansion_GEE>.

The readme.md file within this repository contains commented code for each of the steps and example outputs where appropriate. Javascript (.js) versions of each of the coding elements are also shared on the above GitHub page. Google Earth Engine version XXX was used for this work. We would be grateful if all those who use the shared code could reference or cite this manuscript in any future work.

**Reference**

Roy DP, Kovalskyy V, Zhang HK, Vermote EF, Yan L, Kumar SS, Egorov A (2016) Characterization of Landsat-7 to Landsat-8 reflective wavelength and normalized difference vegetation index continuity. Remote Sensing of Environment*,* **185**, 57-70.
